# GC-MS Profiling of Volatile Components in Different Fermentation Products of *Cordyceps Sinensis* Mycelia

**DOI:** 10.3390/molecules22101800

**Published:** 2017-10-24

**Authors:** Hongyang Zhang, Yahui Li, Jianing Mi, Min Zhang, Yuerong Wang, Zhihong Jiang, Ping Hu

**Affiliations:** 1 School of Chemistry and Molecular Engineering, East China University of Science and Technology, Shanghai 200237, China; hongyang_zhang@ecust.edu.cn (H.Z.); yahuili1990@163.com (Y.L.); wangyuerong@ecust.edu.cn (Y.W.); 2 Shanghai Key Laboratory of New Drug Design, School of Pharmacy, East China University of Science and Technology, Shanghai 200237, China; zhangm@ecust.edu.cn; 3 State Key Laboratory of Quality Research in Chinese Medicine, Macau Institute for Applied Research in Medicine and Health, Macau University of Science and Technology, Taipa, Macau 999078, China; mjnrhw@hotmail.com (J.M.); zhjiang@must.edu.mo (Z.J.)

**Keywords:** *Cordyceps sinensis* mycelia, fermentation products, volatile profiling, gas chromatography-mass spectrometry, Simultaneous distillation-extraction, partial least squares-discriminant analysis, quality evaluation

## Abstract

The fermentation products of *Cordyceps sinensis* (*C. sinensis*) mycelia are sustainable substitutes for natural *C. sinensis*. However, the volatile compositions of the commercial products are still unclear. In this paper, we have developed a simultaneous distillation-extraction (SDE) and gas chromatography-mass spectrometry (GC-MS) method for the profiling of volatile components in five fermentation products. A total of 64, 39, 56, 52, and 44 components were identified in the essential oils of Jinshuibao capsule (JSBC), Bailing capsule (BLC), Zhiling capsule (ZLC), Ningxinbao capsule (NXBC), and Xinganbao capsule (XGBC), respectively. 5,6-Dihydro-6-pentyl-2H-pyran-2-one (massoia lactone) was first discovered as the dominant component in JSBC volatiles. Fatty acids including palmitic acid (C16:0) and linoleic acid (C18:2) were also found to be major volatile compositions of the fermentation products. The multivariate partial least squares-discriminant analysis (PLS-DA) showed a clear discrimination among the different commercial products as well as the counterfeits. This study may provide further chemical evidences for the quality evaluation of the fermentation products of *C. sinensis* mycelia.

## 1. Introduction

*Cordyceps sinensis* (*C. sinensis*), a parasitic complex of fungus and caterpillar (“winter worm summer grass”), is a unique and precious medicinal herb in China [1]. It has long been used as a tonic food and enjoyed an extensive praise for its medicinal functions to replenish the kidney and soothe the lung [2]. Modern pharmacological studies also showed that *C. sinensis* was beneficial to the circulatory, immune, hematogenic, cardiovascular, respiratory, and glandular systems in human body [3,4]. However, the natural *C. sinensis* is extremely expensive because it is only found in the prairie soil at an elevation of 3500–5000 m in Western China [5]. Due to the limited distribution, high price, and excessive exploitation, the resource of natural *C. sinensis* has no longer adequate for human need. Therefore, much effort should be spent on discovering sustainable substitutes.

Currently, a number of mycelial strains have been isolated from natural *C. sinensis* and manufactured in large quantity by fermentation technology [6]. The fermented *C. sinensis* mycelia were generally accepted to have similar functions as the natural herbs and are commonly sold as authenticated products in the area of Eastern Asia [7]. In the Chinese market, there are several famous fermentation products of *C. sinensis* mycelia, such as Jinshuibao capsule (JSBC), Bailing capsule (BLC), Zhiling capsule (ZLC), Ningxinbao capsule (NXBC), and Xinganbao capsule (XGBC) [8]. JSBC is prepared by the submerged fermentation of *Paecilomyces hepiali* (strain Cs-4); BLC and ZLC are fermented from *Hirsutella sinensis* (strain Cs-C-Q80) and *Mortierella* SP, respectively; NXBC and XGBC are the mycelial products produced from *Cephalosporium sinensis* and *Gliocladium roseum*, respectively [9]. Since these commercial products are cultivated from different mycelial species, they may possess some different active components and pharmacological effects.

Previous studies have reported many bioactive constituents in natural and cultured *C. sinensis*, such as nucleosides (adenosine, inosine, and cordycepin), carbohydrates (mannitol, trehalose, and polysaccharides), and sterols (ergosterol), etc. [10,11,12]. In contrast, the volatile components in *C. sinensis* were seldom evaluated due to the absence of references or less understanding on their pharmacological activities [13]. Although several free fatty acids and sterols have recently been analyzed, there is still a lack of comprehensive volatile profiling of *C. sinensis* as well as its fermentation products [14]. Actually, the fermentation products cultivated from different mycelial strains have their characteristic odors. For example, JSBC exudes the special aroma of lactones, while BLC and XGBC give off the insect or burning smells, respectively. These suggest that there are differences of volatile compositions between the essential oils of different strains, which may also contribute to the effects of *C. sinensis* products.

In this work, a gas chromatography-mass spectrometry (GC-MS) method for the profiling of volatile components in the fermentation products of *C. sinensis* mycelia was developed. The essential oils of the products were extracted using simultaneous distillation-extraction (SDE). Qualitative analysis was performed by comparing the mass spectra with the library and confirmed by their retention indices and fragmentation patterns. In addition, five commercial products of JSBC, BLC, ZLC, NXBC, and XGBC along with three counterfeits were comparatively analyzed and differentiated using this method combined with multivariate partial least squares-discriminant analysis (PLS-DA).

## 2. Results and Discussion

### 2.1. SDE Extraction of Essential Oils

**Figure 1:**
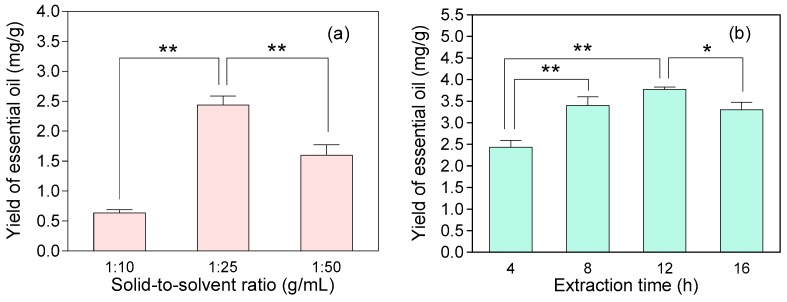
Yields of essential oil (mg oil/g dried material) of Jinshuibao capsule (JSBC) product extracted by the distillation-extraction (SDE) method under different conditions. (**a**) Changes in solid-to-solvent ratio (g/mL) while the extraction time was fixed at 4 h; (**b**) changes in extraction time (h) while the solid-to-solvent ratio was fixed at 1:25 g/mL. The data are represented as mean ± SD (*n* = 3 in each analysis) with * *p* < 0.05 and ** *p* < 0.01.

Compared with conventional techniques (such as solvent extraction and hydrodistillation), the SDE method combines the advantage of liquid-liquid and steam distillation extraction, which ensures obtaining a wider volatile profile of essential oils with high recoveries [15]. In this experiment, parallel extractions of the essential oils of JSBC were carried out using the SDE method as compared to the hydrodistillation method recorded in Chinese Pharmacopoeia [16]. The yields of essential oils (mg oil/g dried material) extracted by the hydrodistillation method and SDE method were between 1–2 mg/g and 2–3 mg/g, respectively, which indicated that the latter method was more efficient. Furthermore, the effects of solid-to-solvent ratio and extraction time in the SDE method were investigated using univariate analysis. The optimal condition with highest yield was obtained at solid-to-solvent ratio of 1:25 g/mL and time period of 12 h (Figure 1). The essential oil yields of different fermentation products extracted by the optimized SDE method are shown in Table 1.

As seen in Table 1, there were obvious differences in the essential oil contents of different fermentation products. The average yield of essential oil in JSBC (3.0 mg/g) was much higher than in the other products, with the average yields produced variously between 0.7–1.0 mg/g. This may be due to the different mycelial species and fermentation processes of these products. The relative standard deviations (RSDs) of each batch of products listed in Table 1 were less than 8.6%, which indicated that the stabilities of the manufacturing fermentation technologies were satisfactory. Therefore, the yields of essential oil could be considered as candidate indicators for the quality assessment of the fermentation products of *C. sinensis* mycelia.

### 2.2. GC-MS Volatile Profiling and Method Validation

**Figure 2:**
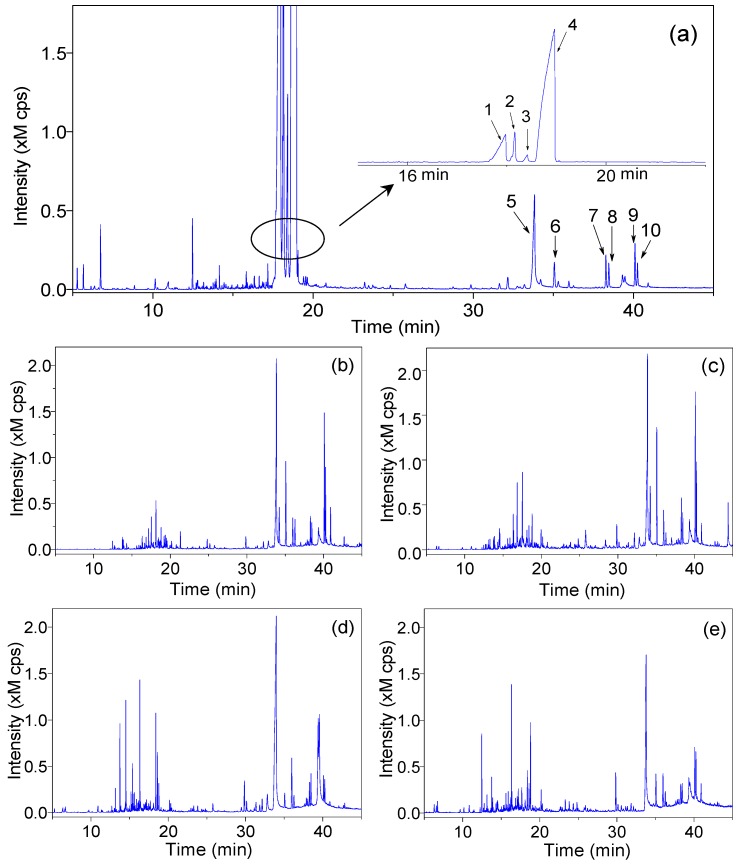
Total ion chromatograms (TICs) of essential oils in different fermentation products of *C. sinensis*. (**a**) JSBC; (**b**) Bailing capsule (BLC); (**c**) Zhiling capsule (ZLC); (**d**) Ningxinbao capsule (NXBC); and (**e**) Xinganbao capsule (XGBC). The numbered peaks in chromatogram of JSBC are the common peaks for method validation test.

**Figure 3:**
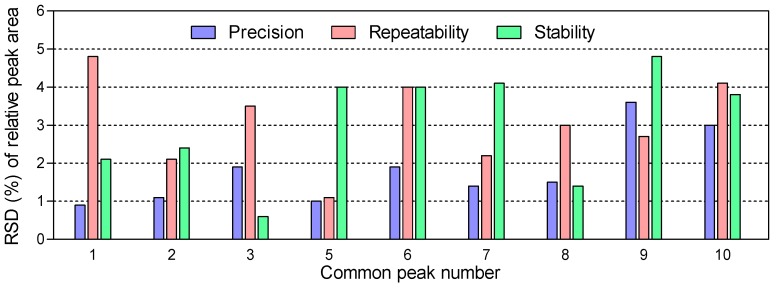
The relative standard deviations (RSDs) of relative peak areas of the common peaks in precision, repeatability, and stability tests. The relative peak area is the ratio of peak area of each peak to the reference peak (peak 4 of massoia lactone, as seen in Figure 4). The peak numbers are consistent with those in Figure 2a.

The instrument parameters, including the flow rate, split ratio, and temperature programming, were investigated to obtain the optimal separation and detection conditions. The total ion chromatogram (TIC) of representative essential oil extract of JSBC is showed in Figure 2a. Peak 4 (massoia lactone) was assigned as the reference peak for its highest content and important pharmaceutical actions (as described in Section 2.3). The relative peak areas of all common peaks to this reference peak were then calculated. The overall RSDs of relative peak areas of the common peaks in precision, repeatability, and stability tests were less than 3.6%, 4.8%, and 4.8%, respectively (Figure 3). The proposed GC-MS method is therefore acceptable for the volatile profiling of fermentation products.

The TICs of essentials oils extracted from other products are shown in Figure 2b–e. It could be observed that the volatiles of JSBC were mainly composed of low-boiling components (eluted before 25 min in Figure 2a), while the other products were found to have more high-boiling components (eluted after 25 min in Figure 2b–e) than JSBC. These results indicated distinct variations of the volatile compositions between JSBC and the other products.

### 2.3. Identification of Volatile Components in Fermentation Products

The peaks in TICs were identified by matching their mass spectra with those of reference compounds recorded in NIST MS library and confirmed by the Kovats retention index (RI) obtained from a series of *n*-alkanes [17,18,19,20,21,22]. The qualitative data of volatile components in different fermentation products with their peak area percentages are presented in Table 2. A total of 64, 39, 56, 52, and 44 compounds were identified in JSBC, BLC, ZLC, NXBC, and XGBC, accounting for 81.98%, 78.85%, 76.84%, 77.80%, and 73.66% of the total peaks areas of essential oils, respectively. In general, the identified compounds mainly included lactones, fatty acids, aldehydes, ketones, alcohols, phenols, pyrazines, and hydrocarbons, but the contents of these components varied greatly among the five different products (Table 2).

**Figure 4:**
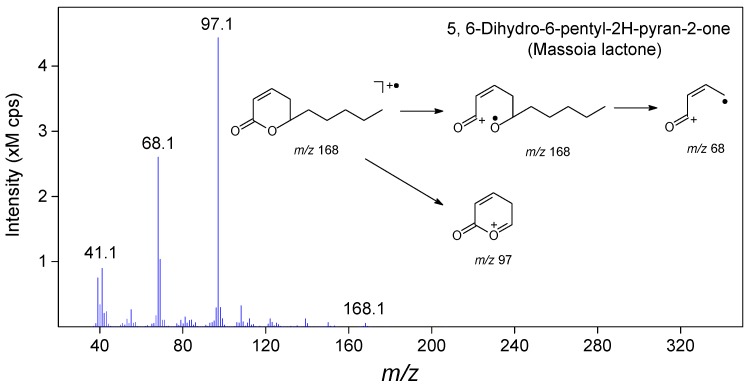
Mass spectrum and fragmentation pathways of massoia lactone (No. 48 in Table 2).

It is worth noting that, the compounds of 5,6-dihydro-6-pentyl-2*H*-pyran-2-one (massoia lactone, No. 48 in Table 2) and its analogue 5,6-dihydro-6-propyl-2*H*-pyran-2-one (No. 41 in Table 2) [23], were first identified in the fermentation products of *C. sinensis* mycelia. Here we take massoia lactone as an example to illustrate the identification process. Firstly, by using mass spectra matching, the NIST MS library provided a reliable searching result of massoia lactone with a high matching score of 90. Examination of the mass spectrum (Figure 4) showed the molecular ion of massoia lactone at *m/z* 168. The most abundant ion at *m/z* 97 was assigned to be the characteristic fragment corresponding to the α-cleavage of *n*-amyl side chain. Analysis of the less abundant ion at *m/z* 68 revealed the fragment generated from the aromatic ring-cleavage and subsequent rearrangement occurred in the β-unsaturated lactone. In addition, the RI value of target peak was calculated as 1476 in this experiment, which was consistent with the value of massoia lactone reported in the literature (RI = 1474) [24]. To confirm this identification, isolation and NMR analysis of the target component were performed and the data were listed as follows: isolated as a colorless oil (compound purity > 95%); ^1^H NMR *δ* 6.96–6.72 (m, 1H, CH=CHCH_2_), 6.02 (d, *J* = 9.9 Hz, 1H, CH=CHCH_2_), 4.53–4.20 (m, 1H, CH), 2.41–2.23 (m, 2H, CH=CHCH_2_), 1.88–1.33 (m, 8H, 4CH_2_), 0.90 (t, *J* = 6.7 Hz, 3H, CH_3_); ^13^C NMR *δ* 164.41 (C=O), 145.10 (CH=CH), 121.02 (CH=CH), 77.82 (CH_2_CHO), 34.56 (CH=CHCH_2_), 31.27 (CH_2_), 29.13 (CH_2_), 24.23 (CH_2_), 22.23 (CH_2_), 13.72 (CH_2_) (Figures S1 and S2). The GC-MS spectra and retention times of the purified compound were also verified with those of JSBC volatiles. All above information is enough for the confirmation of this compound as massoia lactone [25].

**Figure 5:**
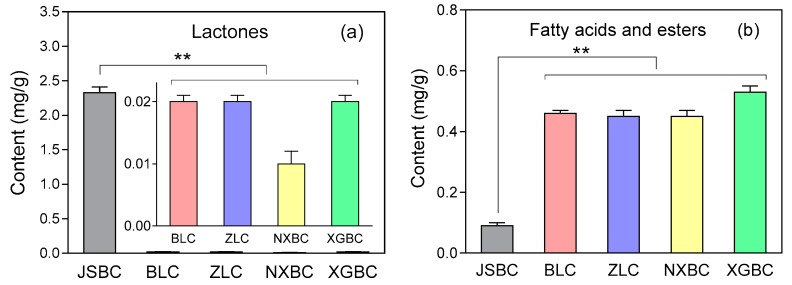
Contents (mg component/g dried material) of (**a**) lactones and (**b**) fatty acids and esters in different fermentation products of *C. sinensis*. The data are represented as mean ± SD (*n* = 3 in each group) with ** *p* < 0.01.

As seen in Table 2, several lactones (mainly massoia lactone) were found in the essential oils of JSBC, BLC, ZLC, NXBC, and XGBC, accounting for 77.70%, 2.71%, 2.58%, 1.80%, and 1.61% of the total peak areas, respectively. After considering the yields of essential oil, the contents of total lactones (mg component/g dried material) in each fermentation product were calculated. As seen in Figure 5a, the content of lactones in JSBC was much higher than in other products. Additionally, massoia lactone was the dominant component in JSBC volatiles (accounting for 77.46% of the total peak areas) and thus could be regarded as a marker for quality control of this product (Table 2). Massoia lactone is a fragrant agent that may be sensed as coconut, cream or butter, and is therefore used in food industry as a flavor additive [26]. Recent studies suggest that massoia lactone exhibits potential antifungal, antivirus, anticancer, or anti-inflammatory activities, which might contribute to the pharmacological effects of JSBC [25,27].

The fatty acids and their esters were another major components presented in the essential oils of JSBC, BLC, ZLC, NXBC, and XGBC, accounting for 2.99%, 65.09%, 64.99%, 63.88%, and 52.74% of the total peak areas, respectively. The contents of fatty acids and esters (mg component/g dried material) in each fermentation product were then calculated considering the yields of essential oil. The fatty acids and esters were the most abundant components in the volatiles of BLC, ZLC, NXBC, and XGBC, and their contents were found to be significantly higher in the four products than in JSBC (Figure 5b). As seen in Table 2, palmitic acid (C16:0) and linoleic acid (C18:2) were the main fatty acids in the fermentation products, which was corresponded to the previous report for cultured *C. sinensis* [14]. Free fatty acids are a group of essential nutrients and bioactive components, possessing a wide range of pharmacological actions including antioxidant, cardioprotective, and nephroprotective effects [28,29,30].

### 2.4. Multivariate PLS-DA Analysis

**Figure 6:**
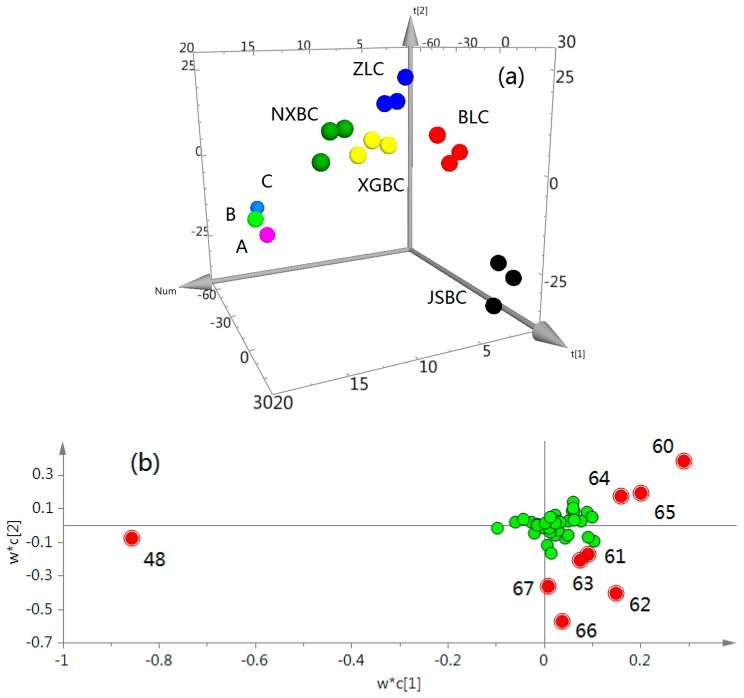
PLS-DA scores plot (**a**) and loadings plot (**b**) of different fermentation products of *C. sinensis*. JSBC (black, *n* = 3); BLC (red, *n* = 3); ZLC (blue, *n* = 3); NXBC (green, *n* = 3); XGBC (yellow, *n* = 3); Counterfeit product A (pink), B (bright green), and C (light blue). The numbers of potential marker components (indicated in red) in loadings plot are consistent with those in Table 2.

Using the method described above, GC-MS analysis of the essential oils extracted from five fermentation products (*n* = 3 in each group) and three counterfeits were undertaken, respectively. A supervised PLS-DA method was then applied to visualize the variations among these samples. The first three components accounted for 42.7%, 22.2%, and 7.31% of total variances, respectively, which indicated that the model was reliable. Clear discrimination of different groups was observed in the PLS-DA scores plot where each point represents an individual sample (Figure 6a). The tight clusters of samples in each group (except the counterfeits) demonstrated that the stabilities of the manufacturing fermentation technologies were guaranteed. As seen in Figure 6a, the JSBC group was found to be far away from the other groups, reflecting significant differences of the volatile compositions between JSBC and other products. Furthermore, the scores plot showed a distinct separation of the three counterfeits from other authenticated commercial products. Therefore, our proposed GC-MS method combined with PLS-DA analysis is helpful for evaluating the volatile compositions and thus quality of the fermentation products, especially for the counterfeits identification.

In the corresponding loadings plot (Figure 6b), the distance of individual variables from the main cluster is positively related to their influence on the group separation, that means the compounds (variables) far away from the main cluster have greater impact on the classification. Moreover, the VIP (variable importance in the projection) values of each compound were calculated. The compounds with lager VIP values represent higher contributions to the discrimination of different groups. Finally, nine volatile components with VIP values ≥1.00 were selected as potential markers, including massoia lactone (No. 48 in Table 2), fatty acids of palmitic acid and linoleic acid (No. 60 and 65 in Table 2), and other fatty acid esters. This finding is consistent with the GC-MS profiling results and the identified marker components will be useful for distinguishing the different fermentation products.

## 3. Materials and Methods

### 3.1. Reagents and Materials

Analytical grade anhydrous ether, anhydrous sodium sulfate, sodium chloride, petroleum ether, dichloromethane, and methanol were obtained from Shanghai Lingfeng Chemical Reagent Company (Shanghai, China). *N*-alkanes (C7-C40) were purchased from Sigma-Aldrich (St. Louis, MO, USA). Ultrapure water (18.2 MΩ) was purified with an EPED-E2-10TF water purification system (EPED Technology Development Co., Ltd., Nanjing, China). Five commercial fermentation products of JSBC (Jiminkexin Pharmaceutical Company, Yichun, Jiangxi, China), BLC (Zhongmei Pharmaceutical Company, Hangzhou, Zhejiang, China), ZLC (Tianyuan Pharmaceutical Company, Hangzhou, Zhejiang, China), NXBC (Zhengdaqingchunbao Pharmaceutical Company, Hangzhou, Zhejiang, China), and XGBC (Changtian Pharmaceutical Company, Baoding, Hebei, China) were purchased from local drugstore. The three unauthorized counterfeit fermentation products (marked as A, B, and C) were obtained from the local market.

### 3.2. Sample Preparation

10 g samples of fermentation products were immersed in a round-bottom flask with 250 mL of ultrapure water, and 50 mL of anhydrous ether was applied as solvent in another flask. Both flasks were placed in a Likens-Nickerson apparatus (Changcheng Glass Instrument Factory, Gaoyou Lake Tianchang City, Anhui, China) and heated up to their boiling points. The distillation extraction was continued for 12 h. After then, the extract was collected at room temperature and dried over anhydrous sodium sulfate. The extract was evaporated under nitrogen and weighed for calculating the essential oil yields of different products. The dried extract was finally re-dissolved in 1 mL of anhydrous ether before GC-MS analysis. According to the results of Table 1, approximately 7–30 mg essential oils could be extracted during the procedure.

### 3.3. GC-MS Analysis

GC-MS analysis was performed on an Agilent 7890A gas chromatography instrument coupled to an Agilent 5975C quadrupole mass spectrometer (Santa Clara, CA, USA). A HP-5MS capillary column (30 m × 0.25 mm i.d., 0.25 µm film thickness, Varian Inc., Palo Alto, CA, USA) was used for separation. The temperature program was set as following: the initial oven temperature was set at 40 °C and held for 2 min, then programmed at 5 °C/min to 70 °C and held for 2 min, then at 15 °C/min to 160 °C and held for 5 min, then at 2 °C/min to 180 °C and held for 2 min, and finally at 5 °C/min to 280 °C and held for 2 min. High purity helium (99.999%) was used as carrier gas at a flow rate of 1.0 mL/min. The injection volume was 1 µL with a split ratio of 1:100, and injector temperature was set at 280 °C. The mass spectrometer was operated in electron impact (EI) mode with the ionization energy of 70 eV. Full mass scan of 35–480 amu was used and the scan rate was 0.32 s per scan. The temperatures of quadrupole and ion source were kept at 150 °C and 230 °C, respectively. Qualitative analysis was performed by the National Institute of Standards and Technology (NIST 11) MS library.

### 3.4. Analytical Method Validation

To ensure the reproducibility and stability of the method, validation tests were performed with a set of JSBC samples according to the routine procedures [31]. Precision of injection was carried out by five replicated analyses of the same sample. Five parallel samples were prepared using the same protocol and analyzed to examine the reproducibility of the method. To test the stability, the same sample was analyzed at five different time points (0, 4, 8, 16, and 24 h) within one day. Variations of each test were evaluated by calculating the RSDs of relative peak areas of nine common peaks in the chromatogram (numbered in Figure 2a). The relative peak area is the ratio of peak area of each peak to the reference peak (peak 4 of massoia lactone, as seen in Figure 4).

### 3.5. Isolation and Characterization of Massoia Lactone

0.5 g essential oil of JSBC product (extracted as described in Section 3.2) were dissolved in petroleum ether and subjected to a silica-gel column. The samples were eluted in turn with petroleum ether, petroleum ether/dichloromethane (50:50, *v*/*v*), and methanol, respectively, and the eluents were collected and monitored by TLC spotting. The high purity fractions of massoia lactone were combined. After evaporation of the solvent under reduced pressure, the residue was frozen and lyophilized overnight. ^1^H (400 MHz) and ^13^C (100 MHz) NMR spectra were recorded in deuterated chloroform with tetramethylsilane as internal standard on an Agilent 400-MR NMR spectrometer (Palo Alto, CA, USA).

### 3.6. Chemometric Analysis

A report of the identified volatile components (listed in Table 2) in each product with their peak area percentages were generated in CSV format. The resulting 3D dataset, including compound names (variables), sample names (observations), and relative contents, were imported into SIMCA P+ 13.0 software (Umertrics, Umea, Sweden). Pareto-scaled and mean-centered pretreatments of the dataset were performed before multivariate analysis. PLS-DA analysis was applied to visualize the clustering among groups and identify the differentially changed components responsible for the separation.

## 4. Conclusions

In the current study, a method for the profiling of volatile components in the fermentation products of *C. sinensis* mycelia was developed by using SDE and GC-MS analysis. Under optimized conditions, a total of 64, 39, 56, 52, and 44 compounds were identified in the essential oils of JSBC, BLC, ZLC, NXBC, and XGBC, respectively. Massoia lactone was discovered as the dominant component in JSBC volatiles and thus could be considered as a marker for quality control of this product. In contrast, fatty acids and their esters were found to be the most abundant volatile compositions of the other four products. The PLS-DA results also demonstrated that the above components contributed more to the separation of different commercial products as well as the counterfeits. This analytical method combined with multivariate analysis should be helpful for the quality evaluation of the fermentation products of *C. sinensis* mycelia.

## Figures and Tables

**Table 1:** Yields of essential oil in different fermentation products of *C. sinensis* ^1^.

Samples	Batch No.	Yields ^2^	Average Yield	RSD%
JSBC-1	131004	2.9		
JSBC-2	130913	2.8	3.0	7.0
JSBC-3	140208	3.2		
BLC-1	121243	0.7		
BLC-2	130749	0.8	0.7	7.8
BLC-3	131128	0.7		
ZLC-1	130406	0.7		
ZLC-2	130703	0.7	0.7	7.8
ZLC-3	130902	0.8		
NXBC-1	1401001	0.6		
NXBC-2	1401003	0.7	0.7	8.6
NXBC-3	1306002	0.7		
XGBC-1	130407	1.0		
XGBC-2	18130101	1.1	1.0	5.6
XGBC-3	18140104	1.0		

^1^ Yields of essential oil are expressed as mg oil/g dried material; ^2^ the data were obtained by using the optimized SDE method.

**Table 2:** Volatile Components identified in different fermentation products of *C. sinensis*.

No.	*t*_R_ (min)	Compound ^1^	Class	Formula	Average Peak Area Percentage (%, *n* = 3)					RI ^2^
					JSBC	BLC	ZLC	NXBC	XGBC	
**1**	5.28	Hexanal	Aldehyde	C_6_H_12_O	0.06	-	-	0.16	-	801 ^3^
**2**	6.13	Furfural	Aldehyde	C_5_H_4_O_2_	0.01	-	-	-	-	829 ^3^
**3**	6.36	3-Methylbutanoic acid	Fatty acid	C_5_H_10_O_2_	0.02	-	0.21	0.35	0.59	831 ^3^
**4**	6.60	2-Methylbutanoic acid	Fatty acid	C_5_H_10_O_2_	0.01	-	0.20	0.45	0.43	841 ^3^
**5**	6.73	2-Furanmethanol	Alcohol	C_5_H_6_O_2_	0.22	-	0.19	0.16	0.50	853 ^3^
**6**	7.77	2-Heptanone	Ketone	C_7_H_14_O	0.01	-	-	-	-	895 ^3^
**7**	8.09	Heptanal	Aldehyde	C_7_H_14_O	0.01	-	-	-	-	901 ^3^
**8**	10.14	Benzaldehyde	Aldehyde	C_7_H_6_O	0.05	-	-	-	0.30	964 ^3^
**9**	10.29	5-Methylfurfural	Aldehyde	C_6_H_6_O_2_	0.01	-	-	-	-	978 ^3^
**10**	10.95	Hexanoic acid	Fatty acid	C_6_H_12_O_2_	0.07	-	0.27	0.33	0.50	982 ^3^
**11**	11.37	2-Ethyl-5-methylpyrazine	Pyrazine	C_7_H_10_N_2_	-	-	0.08	0.07	-	993 ^3^
**12**	11.44	2-Ethyl-6-methylpyrazine	Pyrazine	C_7_H_10_N_2_	0.01	-	0.08	-	-	1003 ^3^
**13**	11.47	2,3,5-Trimethylpyrazine	Pyrazine	C_7_H_10_N_2_	0.01	-	-	-	-	1005 ^3^
**14**	12.26	Benzyl alcohol	Alcohol	C_7_H_8_O	0.01	-	0.08	0.05	0.18	1035 ^3^
**15**	12.70	Phenylacetaldehyde	Aldehyde	C_8_H_8_O	0.20	0.65	0.17	0.17	2.92	1049 ^3^
**16**	12.80	2-Acetylpyrrole	Pyrrole	C_6_H_7_NO	0.05	0.20	0.16	0.06	0.34	1055 ^3^
**17**	12.93	Acetophenone	Ketone	C_8_H_8_O	0.01	-	0.12	0.10	-	1064 ^3^
**18**	13.08	p-Cresol	Phenol	C_7_H_8_O	0.01	0.18	0.29	0.10	-	1072 ^3^
**19**	13.16	2,5-Dimethyl-3-ethylpyrazine	Pyrazine	C_8_H_12_N_2_	0.02	-	0.15	0.58	0.56	1082 ^3^
**20**	13.18	1-Ethenyl-3-ethylbenzene	Hydrocarbon	C_10_H_12_	0.01	0.16	0.28	0.11	-	1084
**21**	13.26	1-Ethenyl-4-ethylbenzene	Hydrocarbon	C_10_H_12_	0.02	0.36	0.25	0.09	0.54	1089
**22**	13.35	2-Methoxyphenol	Phenol	C_7_H_8_O_2_	0.01	-	0.06	-	-	1092 ^3^
**23**	13.51	Undecane	Hydrocarbon	C_11_H_24_	0.01	-	0.06	-	-	1100 ^3^
**24**	13.76	1,3-Diethenylbenzene	Hydrocarbon	C_10_H_10_	0.02	0.62	0.36	1.99	1.27	1114
**25**	13.85	1,3-Dichloro-2-methylbenzene	Aromatic hydrocarbon	C_7_H_6_Cl_2_	0.01	0.33	-	0.10	0.29	1117
**26**	13.86	Phenylethyl alcohol	Alcohol	C_8_H_10_O	0.01	-	-	0.13	-	1119 ^3^
**27**	13.88	1,4-Dichloro-2-methylbenzene	Aromatic hydrocarbon	C_7_H_6_Cl_2_	0.02	0.52	0.55	0.14	0.49	1121
**28**	13.94	4-Nonen-2-one	Ketone	C_9_H_16_O	0.04	-	0.05	-	-	1123
**29**	13.96	1,4-Diethenylbenzene	Hydrocarbon	C_10_H_10_	0.01	0.21	0.13	0.05	0.14	1125
**30**	14.15	3-Nonen-2-one	Ketone	C_9_H_16_O	0.06	-	-	-	-	1132 ^3^
**31**	14.31	1,2-Dichloro-4-methylbenzene	Aromatic hydrocarbon	C_7_H_6_Cl_2_	0.01	0.33	0.24	0.10	0.28	1146
**32**	14.39	2,3-Diethyl-5-methylpyrazine	Pyrazine	C_9_H_14_N_2_	-	-	0.06	0.07	-	1157 ^3^
**33**	14.42	3,5-Diethyl-2-methylpyrazine	Pyrazine	C_9_H_14_N_2_	0.03	-	-	0.09	0.37	1159 ^3^
**34**	14.46	Benzoic acid	Fatty acid	C_7_H_6_O_2_	0.03	-	0.42	0.29	0.54	1162 ^3^
**35**	14.52	4-Ethylphenol	Phenol	C_8_H_10_O	0.04	-	0.51	2.27	0.37	1169 ^3^
**36**	14.55	Octanoic acid	Fatty acid	C_8_H_16_O_2_	0.03	0.44	0.79	0.06	0.48	1171 ^3^
**37**	14.88	2-Decanone	Ketone	C_10_H_20_O	0.01	-	0.12	0.07	-	1209 ^3^
**38**	15.02	2,5-Dimethyl-3-(2-methylpropyl)pyrazine	Pyrazine	C_10_H_16_N_2_	0.02	-	0.16	0.13	0.11	1217
**39**	15.29	2,5-Dimethyl-3-(1-propenyl)pyrazine	Pyrazine	C_9_H_12_N_2_	0.02	-	0.18	0.15	0.24	1238
**40**	15.63	2-Isoamyl-6-methylpyrazine	Pyrazine	C_10_H_16_N_2_	0.02	0.35	0.38	0.45	-	1260
**41**	15.83	5,6-Dihydro-6-propyl-2H-pyran-2-one	Lactone	C_8_H_12_O_2_	0.04	-	0.15	-	-	1275
**42**	15.87	2-Methyl-3-phenyl-2-propenal	Aldehyde	C_10_H_10_O	0.02	0.34	0.32	0.34	0.66	1293 ^3^
**43**	16.07	2-Undecanone	Ketone	C_11_H_22_O	0.01	0.26	0.14	0.19	0.23	1295 ^3^
**44**	16.34	2,4-Decadienal	Aldehyde	C_10_H_16_O	0.05	1.80	1.08	2.59	3.91	1316 ^3^
**45**	16.45	2,5-Dimethyl-3-(3-methylbutyl)pyrazine	Pyrazine	C_10_H_18_N_2_	0.01	0.42	0.14	-	0.24	1329 ^3^
**46**	16.84	Decanoic acid	Fatty acid	C_10_H_20_O_2_	0.03	0.96	2.96	0.18	0.50	1360 ^3^
**47**	16.93	γ-Nonanolactone	Lactone	C_9_H_16_O_2_	0.02	0.49	0.46	0.17	0.13	1364 ^3^
**48**	18.61	5,6-Dihydro-6-pentyl-2*H*-pyran-2-one	Lactone	C_10_H_16_O_2_	77.46	1.78	1.97	1.63	1.48	1476 ^3^
**49**	18.80	5-Methyl-2-phenyl-2-hexenal	Aldehyde	C_13_H_16_O	0.11	1.52	1.72	1.05	4.14	1483
**50**	19.05	δ-Decalactone	Lactone	C_10_H_18_O_2_	0.18	0.44	-	-	-	1492 ^3^
**51**	19.20	Butylated hydroxytoluene	Alcohol	C_15_H_24_O	-	1.93	0.35	-	-	1515
**52**	19.94	Lauric acid	Fatty acid	C_12_H_24_O_2_	0.11	0.93	0.61	0.31	1.08	1557 ^3^
**53**	23.73	2-Pentadecanone	Ketone	C_15_H_30_O	0.01	0.64	0.42	0.24	0.65	1688 ^3^
**54**	25.76	Myristic acid	Fatty acid	C_14_H_28_O_2_	0.05	0.76	1.91	0.19	0.18	1768 ^3^
**55**	27.05	Ethyl myristate	Ester	C_16_H_32_O_2_	-	0.25	0.23	-	-	1809 ^3^
**56**	29.46	Pentadecanoic acid	Fatty acid	C_15_H_30_O_2_	0.03	0.47	0.36	0.38	-	1862 ^3^
**57**	31.14	2-Heptadecanone	Ketone	C_17_H_34_O	0.02	0.23	0.39	0.32	0.58	1902 ^3^
**58**	32.14	Methyl palmitate	Ester	C_17_H_34_O_2_	0.10	0.74	1.17	0.93	0.52	1928 ^3^
**59**	32.76	Palmitoleic acid	Fatty acid	C_16_H_30_O_2_	0.01	1.57	1.70	0.66	0.85	1941 ^3^
**60**	33.82	Palmitic acid	Fatty acid	C_16_H_32_O_2_	1.32	19.55	18.28	32.23	27.07	1969 ^3^
**61**	34.20	Ethyl palmitoleate	Ester	C_18_H_34_O_2_	0.06	4.72	5.21	0.05	0.31	1978 ^3^
**62**	35.07	Ethyl palmitate	Ester	C_18_H_36_O_2_	0.19	7.80	7.92	1.13	2.82	1996 ^3^
**63**	38.28	Methyl linoleate	Ester	C_19_H_34_O_2_	0.19	2.45	2.95	1.56	1.44	2095 ^3^
**64**	38.46	Methyl oleate	Ester	C_19_H_36_O_2_	0.14	1.81	1.81	2.24	1.47	2112 ^3^
**65**	39.36	Linoleic acid	Fatty acid	C_18_H_32_O_2_	0.22	4.12	5.37	17.74	6.07	2138 ^3^
**66**	40.09	Ethyl linoleate	Ester	C_20_H_36_O_2_	0.23	9.57	7.90	2.36	3.40	2163 ^3^
**67**	40.25	Ethyl oleate	Ester	C_20_H_38_O_2_	0.13	5.87	4.49	2.15	3.40	2166 ^3^
**68**	40.92	Ethyl stearate	Ester	C_20_H_40_O_2_	0.02	3.08	1.23	0.29	1.09	2189 ^3^

^1^ Cut-off value of the NIST MS library matching was set at 85 (%); ^2^ Kovats retention index relative to C7-C40 *n*-alkanes on the HP-5MS capillary column; ^3^ Mass spectrum and RI value agreed with the literature data.

## Supplementary Materials

Supplementary materials are available online. Figure S1: ^1^H-NMR spectrum of massoia lactone (No. 48 in Table 2), Figure S2: ^13^C-NMR spectrum of massoia lactone (No. 48 in Table 2).
