# Supplementary material for: GC-MS Profiling of Volatile Components in Different Fermentation Products of Cordyceps Sinensis Mycelia
Source: Molecules. 2017 Oct 24;22(10):1800. doi: 10.3390/molecules22101800 (PMC6151420; doi:10.3390/molecules22101800)
Supplement: Supplementary file 1 [file molecules-22-01800-s001.pdf]

Supplementary materials

GC-MS Profiling of Volatile Components in Different Fermentation Products of *Cordyceps Sinensis* Mycelia

Hongyang Zhang [1,2], Yahui Li [1], Jianing Mi [3], Min Zhang [2], Yuerong Wang [1], Zhihong Jiang [3] and Ping Hu [1,*]

[1] School of Chemistry and Molecular Engineering, East China University of Science and Technology, Shanghai 200237, China; hongyang_zhang@ecust.edu.cn (H.Z.); yahuili1990@163.com (Y.L.); wangyuerong@ecust.edu.cn (Y.W.)
[2] Shanghai Key Laboratory of New Drug Design, School of Pharmacy, East China University of Science and Technology, Shanghai 200237, China; zhangm@ecust.edu.cn
[3] State Key Laboratory of Quality Research in Chinese Medicine, Macau Institute for Applied Research in Medicine and Health, Macau University of Science and Technology, Taipa, Macau 999078, China; mjnrhw@hotmail.com (J.M.); zhjiang@must.edu.mo (Z.J.)
* Correspondence: huping@ecust.edu.cn; Tel.: +86-021-6425-2844

Contents

Figure S1. ^{1}H-NMR spectrum of massoia lactone (No. 48 in Table 2).

Figure S2. ^{13}C-NMR spectrum of massoia lactone (No. 48 in Table 2).

Characterization data of massoia lactone:

Colorless oil (compound purity > 95%); ^{1}H NMR (400 MHz) δ 6.96-6.72 (m, 1H, CH=CHCH$_2$), 6.02 (d, *J* = 9.9 Hz, 1H, CH=CHCH$_2$), 4.53-4.20 (m, 1H, CH), 2.41-2.23 (m, 2H, CH=CHCH$_2$), 1.88-1.33 (m, 8H, 4CH$_2$), 0.90 (t, *J* = 6.7 Hz, 3H, CH$_3$); ^{13}C NMR (100 MHz) δ 164.41 (C=O), 145.10 (CH=CH), 121.02 (CH=CH), 77.82 (CH$_2$CHO), 34.56 (CH=CHCH$_2$), 31.27 (CH$_2$), 29.13 (CH$_2$), 24.23 (CH$_2$), 22.23 (CH$_2$), 13.72 (CH$_2$).

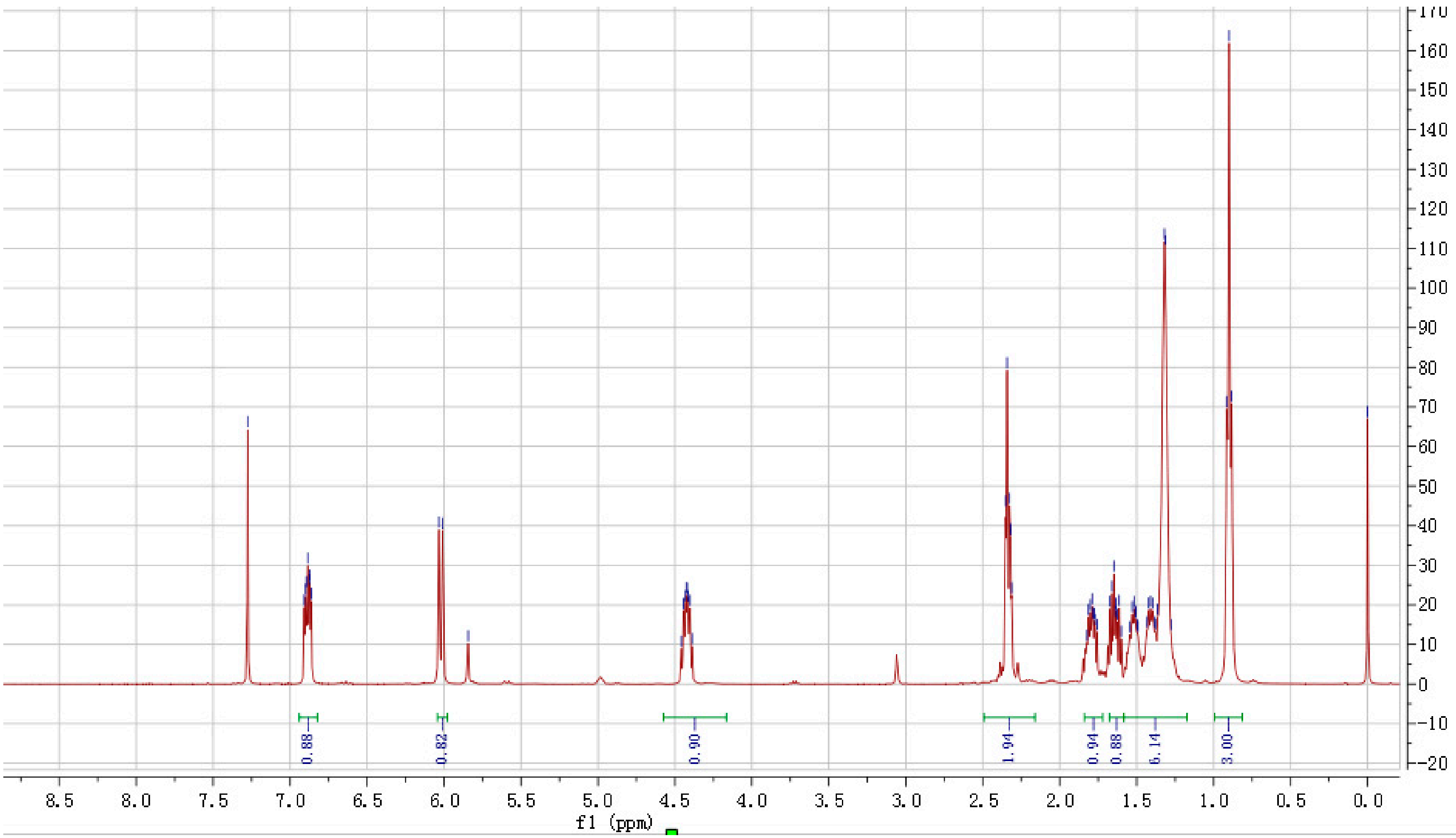

Figure S1. ^{1}H-NMR spectrum of massoia lactone (No. 48 in Table 2).

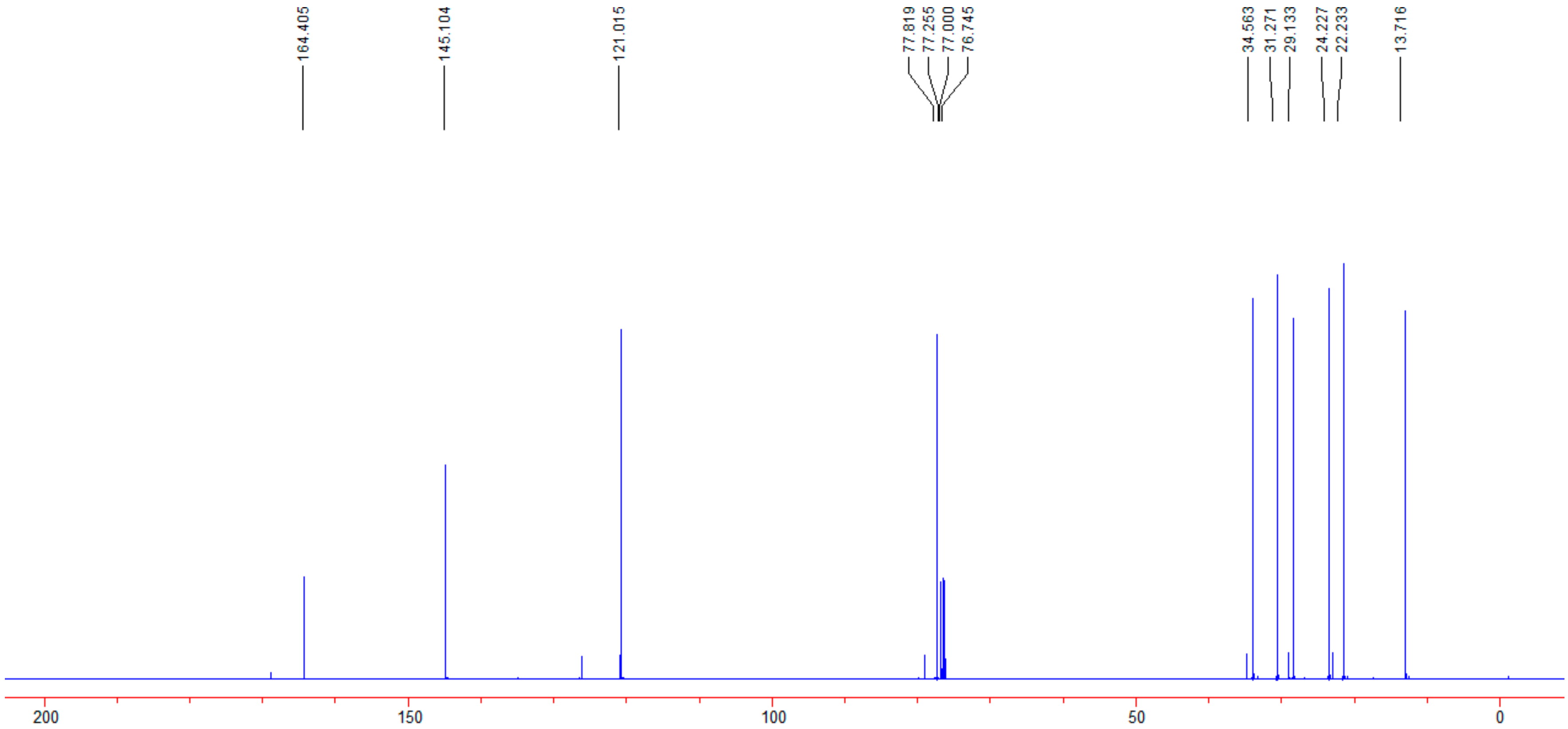

Figure S2. ^{13}C-NMR spectrum of massoia lactone (No. 48 in Table 2).
